# Incorporating sensitive cardiac substructure sparing into radiation therapy planning

**DOI:** 10.1002/acm2.13037

**Published:** 2020-10-18

**Authors:** Eric D. Morris, Kate Aldridge, Ahmed I. Ghanem, Simeng Zhu, Carri K. Glide‐Hurst

**Affiliations:** ^1^ Department of Radiation Oncology University of California – Los Angeles Los Angeles CA USA; ^2^ Department of Radiation Oncology Henry Ford Cancer Institute Detroit MI USA; ^3^ Alexandria Clinical Oncology Department Alexandria University Alexandria Egypt; ^4^ Department of Human Oncology University of Wisconsin – Madison Madison WI USA

**Keywords:** MRI, MRI‐linac, cardiac toxicity, treatment planning

## Abstract

**Purpose:**

Rising evidence suggests that cardiac substructures are highly radiosensitive. However, they are not routinely considered in treatment planning as they are not readily visualized on treatment planning CTs (TPCTs). This work integrated the soft tissue contrast provided by low‐field MRIs acquired on an MR‐linac via image registration to further enable cardiac substructure sparing on TPCTs.

**Methods:**

Sixteen upper thoracic patients treated at various breathing states (7 end‐exhalation, 7 end‐inhalation, 2 free‐breathing) on a 0.35T MR‐linac were retrospectively evaluated. A hybrid MR/CT atlas and a deep learning three‐dimensional (3D) U‐Net propagated 13 substructures to TPCTs. Radiation oncologists revised contours using registered MRIs. Clinical treatment plans were re‐optimized and evaluated for beam arrangement modifications to reduce substructure doses. Dosimetric assessment included mean and maximum (0.03cc) dose, left ventricular volume receiving 5Gy (LV‐V5), and other clinical endpoints. As metrics of plan complexity, total MU and treatment time were evaluated between approaches.

**Results:**

Cardiac sparing plans reduced the mean heart dose (mean reduction 0.7 ± 0.6, range 0.1 to 2.5 Gy). Re‐optimized plans reduced left anterior descending artery (LADA) mean and LADA_0.03cc_ (0.0–63.9% and 0.0 to 17.3 Gy, respectively). LV_0.03cc_ was reduced by >1.5 Gy for 10 patients while 6 cases had large reductions (>7%) in LV‐V5. Left atrial mean dose was equivalent/reduced in all sparing plans (mean reduction 0.9 ± 1.2 Gy). The left main coronary artery was better spared in all cases for mean dose and D_0.03cc_. One patient exhibited >10 Gy reduction in D_0.03cc_ to four substructures. There was no statistical difference in treatment time and MU, or clinical endpoints to the planning target volume, lung, esophagus, or spinal cord after re‐optimization. Four patients benefited from new beam arrangements, leading to further dose reductions.

**Conclusions:**

By introducing 0.35T MRIs acquired on an MR‐linac to verify cardiac substructure segmentations for CT‐based treatment planning, an opportunity was presented for more effective sparing with limited increase in plan complexity. Validation in a larger cohort with appropriate margins offers potential to reduce radiation‐related cardiotoxicities.

## 1 INTRODUCTION

Cardiac toxicity is a major complication of cancer treatment and can occur during, shortly after, and even many years after treatment has been delivered. Long‐term follow‐up of patients undergoing thoracic radiation, such as lymphoma, lung, breast, and esophageal cancers, has shown that in particular, radiation therapy (RT) can lead to radiation‐induced cardiac toxicities such as congestive heart failure, pericardial effusion, coronary artery disease, and myocardial infarction.^1^, ^2^, ^3^

Yet, when a patient’s RT plan is created, only simple whole heart metrics (i.e., mean heart dose (MHD)) are routinely considered for cardiac risk assessment in the current standard of care. The Quantitative Analysis of Normal Tissue Effects in the Clinic (QUANTEC) report assesses dose to the heart as a whole and recommends <10% of it receives >25 Gy as the endpoint of long‐term cardiac mortality.^4^ Importantly, these whole‐heart dose metrics do not provide any information on where dose is distributed.

The heart is a complex organ and dose to its substructures (e.g., coronary arteries, ventricles, atria, great vessels) have been strongly associated with radiation‐induced cardiac morbidity^5^ and future acute coronary events.^6^, ^7^ For example, dose to the left anterior descending artery (LADA) has been linked to an increased risk of myocardial infarction^8^ and development of coronary artery calcifications.^9^ Similarly, higher doses at the base of the heart (i.e., ascending aorta, superior vena cava, and pulmonary artery) are associated with lower rates of patient survival.^10^ Importantly, recent RTOG 0617 subanalyses suggest that dose to the atrial and ventricular cardiac substructures are more strongly associated with survival than assessing dose/volume relationships to the entire heart volume.^11^, ^12^, ^13^ In a recent study by van den Bogaard,^6^ dose to the left ventricular volume receiving 5 Gy predicted major coronary events better than MHD. A study by Hoppe et al. highlighted the importance of quantifying substructure dose as the MHD becomes less correlated to substructure dose with increasingly conformal delivery.^14^ Furthermore, a study by Jacob et al. outlines how the MHD does not accurately predict dose to the left ventricle (LV) and coronary arteries.^15^

**Fig. 1:**
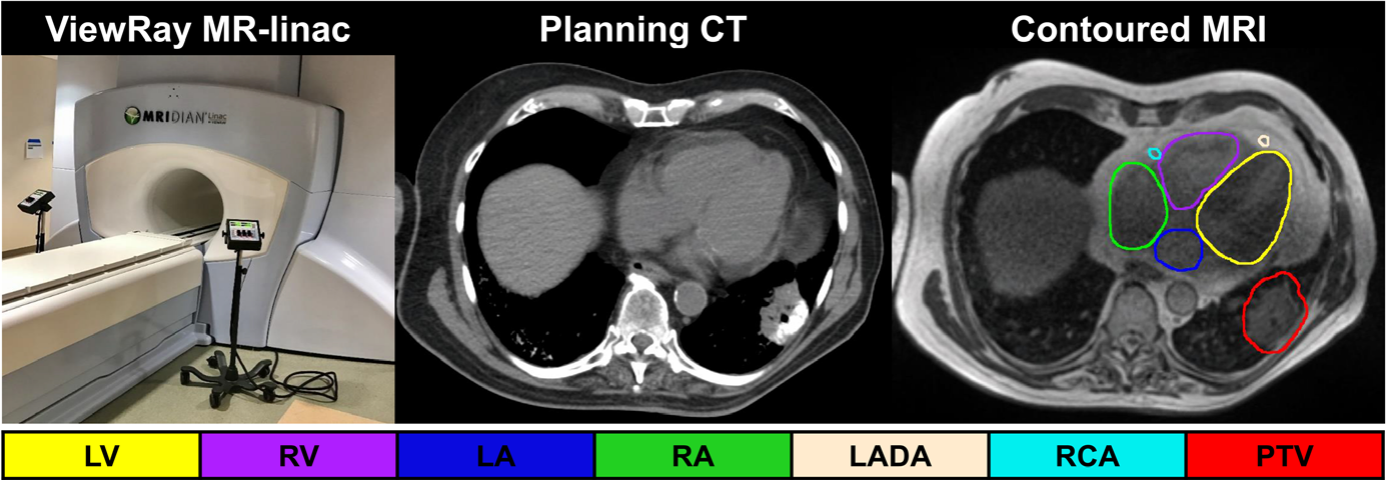
(Left) ViewRay 0.35T MR‐linac, (middle) treatment planning CT, and (right) 0.35T MR dataset with cardiac substructure contours evident and delineated. PTV: planning target volume (malignant neoplasm of lower left lung bronchus). Cardiac‐related abbreviations are defined in the text.

To date, reducing dose to sensitive cardiac substructures has been severely limited because they are not readily visible on standard x‐ray‐based imaging used for both RT planning (i.e., computed tomography simulation (CT‐SIM)) and RT delivery (i.e., cone‐beam CT (CBCT)). Thus, leveraging the superb soft‐tissue contrast of magnetic resonance imaging (MRI) may be advantageous as MRI improves cardiac substructure visibility.^16^, ^17^ Furthermore, the recent introduction of MRI guided linear accelerators (MR‐linacs, Fig. 1 left) has yielded improved tumor and critical structure visualization at 0.35T MRI as compared to CBCT.^18^ MRgRT allows for continuous anatomical visualization of the patient’s heart and target volume throughout treatment which may offer advantages for improved cardiac sparing. Therefore, to advance toward mitigating cardiotoxic side effects from RT, approaches for considering cardiac substructures during treatment planning are urgently needed.

This work sought to apply a multimodality workflow (treatment planning CTs coupled with low‐field MR‐linac MRIs) to integrate sensitive cardiac substructures into treatment planning. This multimodality workflow allowed us to quantify potential dosimetric advantages for improved cardiac sparing through plan re‐optimization and for cases that may benefit, beam angle modifications.

## 2 METHODS

### 2.1 Patient cohort and image acquisition

Fifteen patients with 16 pericardial lesions (i.e., 16 individual plans) who underwent MR‐guided RT for upper thoracic treatments of the lung, mediastinum, and esophagus were retrospectively reviewed on an Institutional Review Board approved study. Of these, 11 were treated with stereotactic body radiation therapy (3–5 fractions to a total dose of 30 to 50 Gy), 2 underwent conventional fractionation (25–35 fractions to a total dose of 50 to 70 Gy), and the remaining 3 were moderately hypofractionated (14–20 fractions to a total dose of 36 to 60 Gy). Patients were imaged in various breathing states (7 end‐exhalation, 7 end‐inhalation, 2 free‐breathing) on a 0.35T ViewRay MRIdian linear accelerator (ViewRay, Mountain View, CA).

All patients were imaged with a balanced steady‐state free precession (bSSFP), TrueFISP acquisition sequence (Siemens, MAGNETOM Avanto, Syngo MR B19) with 15/16 patients with mobile tumors undergoing daily 17–25 sec MRIs (1.5 × 1.5 × 3 mm^3^) under breath‐hold conditions. One patient with a left chest wall lesion could not tolerate breath‐hold and thus underwent a 175‐second free‐breathing MRI for treatment planning. TrueFISP is commonly used in cardiac imaging due to high signal‐to‐noise ratio and imperviousness to motion artifacts.^19^, ^20^ All treatment planning was conducted and dose was calculated on a non‐contrast CT‐SIM in a manner similar to what has been reported for MR‐guided RT of thoracic lesions.^21^ All CT‐SIMs were acquired on a Brilliance Big Bore CT Simulator (Philips Medical Systems, Cleveland, OH) with a 3‐mm slice thickness. MR and CT‐SIM sessions were conducted on the same day and patients were immobilized in the supine position using molded vacuum cushions.

### 2.2 Segmentation and registration

Assessed cardiac substructures included the heart, left/right ventricles (LV, RV), atria (LA, RA), superior/inferior venae cavae (SVC, IVC), ascending aorta (AA), pulmonary artery/veins (PA, PV), left anterior descending artery (LADA), right coronary artery (RCA), and left main coronary artery (LMCA). For 11 patients, a cardiac substructure segmentation atlas^22^ automatically generated the cardiac substructures on the CT‐SIM dataset for treatment planning with the final contours displayed on the low‐field MRI at Fig. 1, right. For the remaining five patients evaluated at a later date, automatic cardiac substructure segmentation on the CT‐SIM was performed using a three‐dimensional U‐Net,^23^ a deep learning model that improved the accuracy and substructure generation time as compared to the atlas method.

While automatic segmentation methods (i.e., multi‐atlas and deep learning methods) provided initial substructure contours on the CT‐SIM datasets, a radiation oncologist consulted the co‐registered low‐field MRI to modify and confirm the final contours used for treatment planning. As shown by the lack of contrast in the planning CT (Fig. 1, center), the enhanced soft tissue contrast from the MRI assisted the generation of more reliable cardiac substructure delineations on the corresponding planning CT. Co‐registration involved an automatic rigid registration based off a manually drawn, local, cardiac confined bounding box. Normalized mutual information was used as the similarity metric as it has been shown to accurately align multimodality images.^24^

### 2.3 Treatment planning

For all patients, the CT‐SIM was used as the primary image set for treatment planning as has been reported in the literature for MRgRT of thoracic lesions.^21^ The co‐registration of the low‐field MR image to the CT‐SIM to elucidate the cardiac substructures was a critical step in allowing the physician to verify the cardiac substructure autosegmentations. Step‐and‐shoot intensity modulated radiation therapy (IMRT) planning was used to generate all 16 RT plans at a dose rate of 600 cGy/min. The MR‐linac utilizes a fast Monte Carlo dose calculation algorithm^25^ and plans were calculated using a 1 × 1 mm dose grid with 1% dose uncertainty.^26^ Plans were prescribed to 95% of the planning target volume with total doses for the original treatment plans varying from 30 to 70 Gy delivered in 4–35 fractions. The original treatment plans for all patients included clinical dose constraints for whole heart endpoints. All clinical treatment plans met physician objectives using standard QUANTEC^27^, ^28^ and TG‐101^29^ dosimetric endpoints for OARs.

Along with adding substructure segmentations retrospectively to the original clinical treatment plans for dose assessment, all plans were re‐optimized to spare cardiac substructures (SPARE plan). Strategies for substructure sparing included evaluating the original plan to identify which cardiac substructures were near the planning target volume (PTV) and thus received the most dose. Optimization objectives were then added with increased priority on the substructures receiving higher doses. If the dose limit was unachievable, constraints were relaxed with the overall objective to minimize substructure dose. If the dose to a particular substructure was minimal in the original plan, an additional objective was added in the IMRT optimization to ensure consistency was maintained.

In addition to adding substructures to the optimization, possible further cardiac sparing improvement was also assessed through modifying the beam arrangement (New Angles plan) after the substructures had already been incorporated into the optimization. For plans with lesions that are particularly close in proximity to the heart, it was evaluated whether beams entering or exiting the heart could be potentially removed or modified to further spare the heart and substructures. IMRT techniques were used for all SPARE and New Angles plans with the substructures integrated into the optimization while maintaining tumor volume coverage and minimizing organ at risk (OAR) dose. Table 1 outlines the dosimetric considerations during plan optimization, derived from the literature, when cardiac substructures were included. All plans were converted to standard fractionation using the equivalent dose to 2 Gy fractions (EQD2, α/β = 2) to allow for uniform evaluation.

**Table 1:** Summary of cardiac substructure sparing objectives utilized in planning optimization for the re‐optimization (SPARE) plan and the New Angles plan.

Substructure	Mean Dose	Maximum Dose	Additional Endpoint
Right ventricle	‐	Minimize^13^	V45^13^
Left ventricle	‐	Minimize^13^	LV‐V5^6^ V45^13^
Left atrium	8.5 Gy^10^ Minimize^13^, ^31^	Minimize^30^	V45^13^
Right atrium	8.5 Gy^10^ Minimize^13^	‐	V45^13^
Superior vena cava	8.5 Gy^10^	‐	D90^30^
PA, PV, AA	8.5 Gy^10^	‐	
Left anterior descending artery	Minimize^32^	< 10 Gy^33^ Minimize	V45^13^
RCA, LMCA	‐	‐	V45^13^

Minimize may be taken as “as low as reasonably achievable” (ALARA). Abbreviations defined in the text.

### 2.4 Dosimetric and statistical assessment

Original, SPARE, and when applicable, New Angle plans were exported from the ViewRay planning system and imported into MIM (version 6.9.4, MIM Software Inc., Cleveland, OH) for automated evaluation. Dosimetric assessment included mean doses, left ventricular volume receiving 5 Gy (LV‐V5), and Dose to 0.03 cc (D_0.03cc_, surrogate for maximum dose) for 12 cardiac substructures and the whole heart. To ensure clinically acceptable plans were still achieved, differences in PTV coverage and dose to the OARs were also assessed. Lastly, total MU and treatment time were evaluated and compared to the original clinical treatment plan as metrics of plan complexity. All dosimetric and planning data were summarized via mean ± standard deviation (SD). As the data were not normally distributed, dosimetric comparisons at each metric were conducted using a two‐tailed Wilcoxon signed ranks test with *P* < 0.05 considered statistically significant. Statistical assessments were conducted in SPSS version 25.0 (SPSS, Chicago, IL).

## 3 RESULTS

### 3.1 Contour generation and plan complexity

The treatment time per fraction (a metric of plan complexity) across the 16 patients after plan re‐optimization was 6.57 ± 3.50 minutes (range 2.60–12.41) for the clinical treatment plan and was 6.93 ± 3.27 minutes (range 2.75–11.99) after re‐optimizing (*P* > 0.05). The mean percent difference in the delivered MUs between the original and re‐optimized plans was 1.7 ± 11.3% (range −21.6 to 15.8%) which did not yield a statistically significant difference (*P* > 0.05).

Four patients benefited from New Angles plans where the number of original treatment beams (range 7–11) shifted by anywhere from −1 to + 3 (range 8–14). For two of the four patients, lesions were directly adjacent to the heart (i.e., a pericardial lymph node and a malignant neoplasm of the lung (Fig. 5)). The other two patients presented with upper lung lobe lesions that were greater than 9 cm away from the heart. The average treatment time for these patients after beam angle modification was 6.12 ± 3.68 minutes, which was not significantly different (*P* > 0.05) from the original treatment time for these 4 patients (6.54 ± 3.31 minutes). Lastly, the mean percent difference in the delivered MUs between the original and re‐optimized plans for these patients was 9.5 ± 16.8% (range −16.6 to 23.8%, *P* > 0.05).

### 3.2 Cardiac Substructure Sparing

The radiation dose to the whole heart after plan re‐optimization met all clinical objectives.^27^, ^28^ All sparing plans significantly reduced the MHD (*P* < 0.05) with an average reduction of 0.7 ± 0.6 Gy (range 0.1 to 2.5 Gy). Furthermore, D_0.03cc_ to the heart was reduced by 8.6 ± 12.1 Gy (range −8.6 to 39.9 Gy) across all patients after plan re‐optimization (*P* < 0.05).

**Fig. 2:**
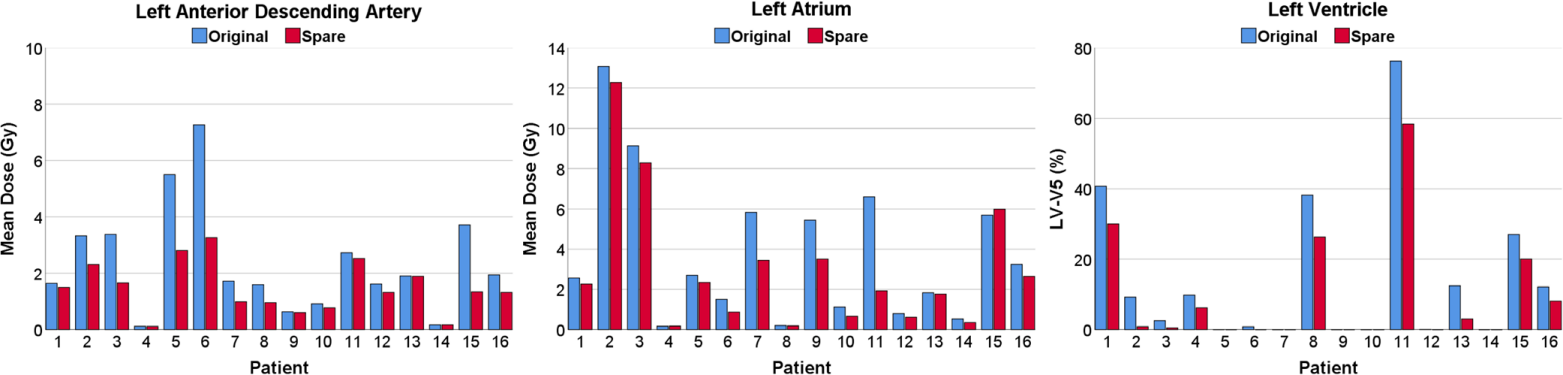
Dose sparing possible by incorporating cardiac substructures into IMRT optimization during MR‐guided radiation therapy planning. The mean dose for all 16 patients is shown for the left anterior descending artery (left) and the left atrium (center). The left ventricular volume receiving 5 Gy (LV‐V5) is shown on the right.

Fig. 2 outlines a subset of dose objectives from Table 1 representing the difference in radiation dose received by the LADA, LA, and LV between the original and clinical treatment plans across all 16 patients. Re‐optimized SPARE plans reduced LADA mean and D_0.03cc_ by anywhere from 0.0 to 4.0 Gy and from 0.0 to 17.3 Gy, respectively (Fig. 2 left). For the 5 patients that had LADA_0.03cc_ doses greater than 10 Gy (threshold for coronary artery calcification^9^ presented in Table 1), 4 were brought below 10 Gy after re‐optimization (average reduction for these patients was 13.4 ± 7.0 Gy). D_0.03cc_ for the remaining patient was reduced from 29.0 to 11.2 Gy. Similarly, D_0.03cc_ to the LV was reduced in 14 cases (range 0.05 to 12.85 Gy) with 10 patients having >1.5 Gy reductions. There was a large reduction (>7%) in LV‐V5 for 6 patients with an initial LV‐V5 greater than 10%. LA mean dose (Fig. 2, center) was either equivalent or reduced (average reduction 0.9 ± 1.2 Gy) for all SPARE plans. For Patient 3, the left atrial mean dose was reduced to <8.5 Gy which has been shown to be a threshold associated with decreased survival,^10^ and highlights the importance of optimizing plans while considering these thresholds. Lastly, the left atrial maximum dose, which has been significantly associated with non‐cancer death,^30^ was reduced by 2.3 ± 6.4 Gy across all 16 patients.

Table 2 summarizes the change in mean dose and D_0.03cc_ to all cardiac substructures. The mean doses to all substructures and the heart were significantly reduced after re‐optimization (*P* < 0.05). Moreover, D_0.03cc_ was significantly reduced after plan re‐optimization in 8 out of 12 substructures, as well as for the whole heart. The LMCA mean and D_0.03cc_ doses were reduced for all patients and were the substructure with the largest reduction in mean dose across all patients (average reduction in LMCA mean dose: 1.13 ± 1.15 Gy). Lastly, the volume of the heart receiving 25 Gy (V25) was significantly reduced on average (n = 11 patients who met the V25 threshold) by 1.08 ± 1.47% (*P* < 0.05).

**Table 2:** Change in D_0.03cc_ and mean dose after plan re‐optimization for the PTV, heart and its substructures, and other organs at risk.

Structure		Average change After re‐optimization	
		Mean dose (Gy)	D_0.03cc_ (Gy) Other clinical endpoint
	PTV	0.37 ± 1.85	1.95 ± 3.67 **PTV95**: 0.03 ± 0.21 Gy
Organs at Risk	Spinal Cord	0.06 ± 0.25	0.38 ± 1.37
	Total lung	−0.01 ± 0.26	1.32 ± 2.70 **V20**: 0.03 ± 0.71 %
	Esophagus	0.25 ± 0.70	0.39 ± 4.06 **V35**: 0.53 ± 2.46 % (n = 5)
Heart and Substructures	Heart	−0.68 ± 0.60*	−8.57 ± 12.06* **V25**: −1.08 ± 1.47* % (n = 11)
	LV	−0.53 ± 0.70*	−3.27 ± 4.08* **LV−V5**: −6.33 ± 5.57* % (n = 12)
	LA	−0.85 ± 1.22*	−2.30 ± 6.42
	RV	−0.55 ± 0.74*	−4.12 ± 4.81*
	RA	−0.52 ± 0.94*	−1.38 ± 4.47
	AA	−0.83 ± 1.13*	−2.23 ± 3.42*
	PA	−0.95 ± 1.60*	−2.84 ± 8.53
	PV	−0.89 ± 1.09*	−2.71 ± 5.69*
	SVC	−0.57 ± 1.19*	−1.08 ± 3.65* **D90**: −0.10 ± 1.23 Gy*
	IVC	−0.16 ± 0.38*	−0.74 ± 1.91
	LADA	−0.91 ± 1.18*	−4.05 ± 5.32*
	LMCA	−1.13 ± 1.15*	−1.31 ± 1.55*
	RCA	−0.65 ± 1.26*	−1.64 ± 3.38*

The asterisk indicates significant reduction in dose after re‐optimization (none of the increases represented here were statistically significant). N = 16 for all structures except for the esophagus where n = 10. For the heart V25, esophagus V35, and LV‐V5, results were reported only for structures with a non‐zero value for the corresponding dosimetric endpoint. Abbreviations are defined in the text.

Further cardiac substructure dose sparing beyond re‐optimization was achieved for 4 patients with beam angle modification where the mean dose reduction across all substructures was 0.6 ± 0.4 Gy (highest mean reduction was in the PA and was 1.5 ± 2.0 Gy). The D_0.03cc_, mean dose, and V25 to the heart were further reduced by 5.4 ± 4.1 Gy, 0.5 ± 0.7 Gy, and 4.2 ± 2.9%, respectively. For the LV, after re‐optimization coupled with beam angle modification, D_0.03cc_ and LV‐V5 were further reduced by 2.1 ± 2.9 Gy and 2.0 ± 1.9%, respectively. Lastly, the SVC D90 improved 3.3 ± 4.0% after the beam angles were modified.

### 3.3 Organs at risk (OARs) and planning target volume (PTV) coverage

All re‐optimized plans met the original clinical prescription dose to the PTV while doses to the OARs met all objectives based on clinically acceptable guidelines.^27^, ^28^ Table 2 outlines the average change in the mean dose and D_0.03cc_ for the PTV and OARs. Across all patients, the esophagus had a negligible change in mean dose after plan re‐optimization (0.25 ± 0.70 Gy, *P* > 0.05). Additionally, differences in clinical endpoints such as the volume of the lung receiving 20 Gy (V20) and volume of the esophagus receiving 35 Gy (V35) were negligible after re‐optimization (*P* > 0.05). No statistically significant changes were observed in the mean dose, D_0.03cc_, and other clinical endpoints for the PTV and OARs (*P* > 0.05). Although the increase in PTV D0.03cc was not statistically significant, target homogeneity may still be decreased a non‐negligible amount due to plan re‐optimization.

For the four patients that benefited from beam angle modification, negligible changes were observed for all of the PTV D95 metrics (range 0 to 0.30 Gy) and three out of four patients’ D_0.03cc_ (<0.5 Gy). However, one patient had an increase in D_0.03cc_ of 3.7%, or 6.2 Gy (EQD2), with beam angle modification when compared to the original clinical treatment plan. Negligible changes (<1%) in clinical endpoints were observed for the esophagus (V35 and V50) and lungs (mean dose and V20) as compared to the original clinical treatment plan while the spinal cord D_0.03cc_ was reduced by 2.3 ± 1.9 Gy with beam angle modification as compared to re‐optimization alone.

### 3.4 Individual patient results

**Fig. 3:**
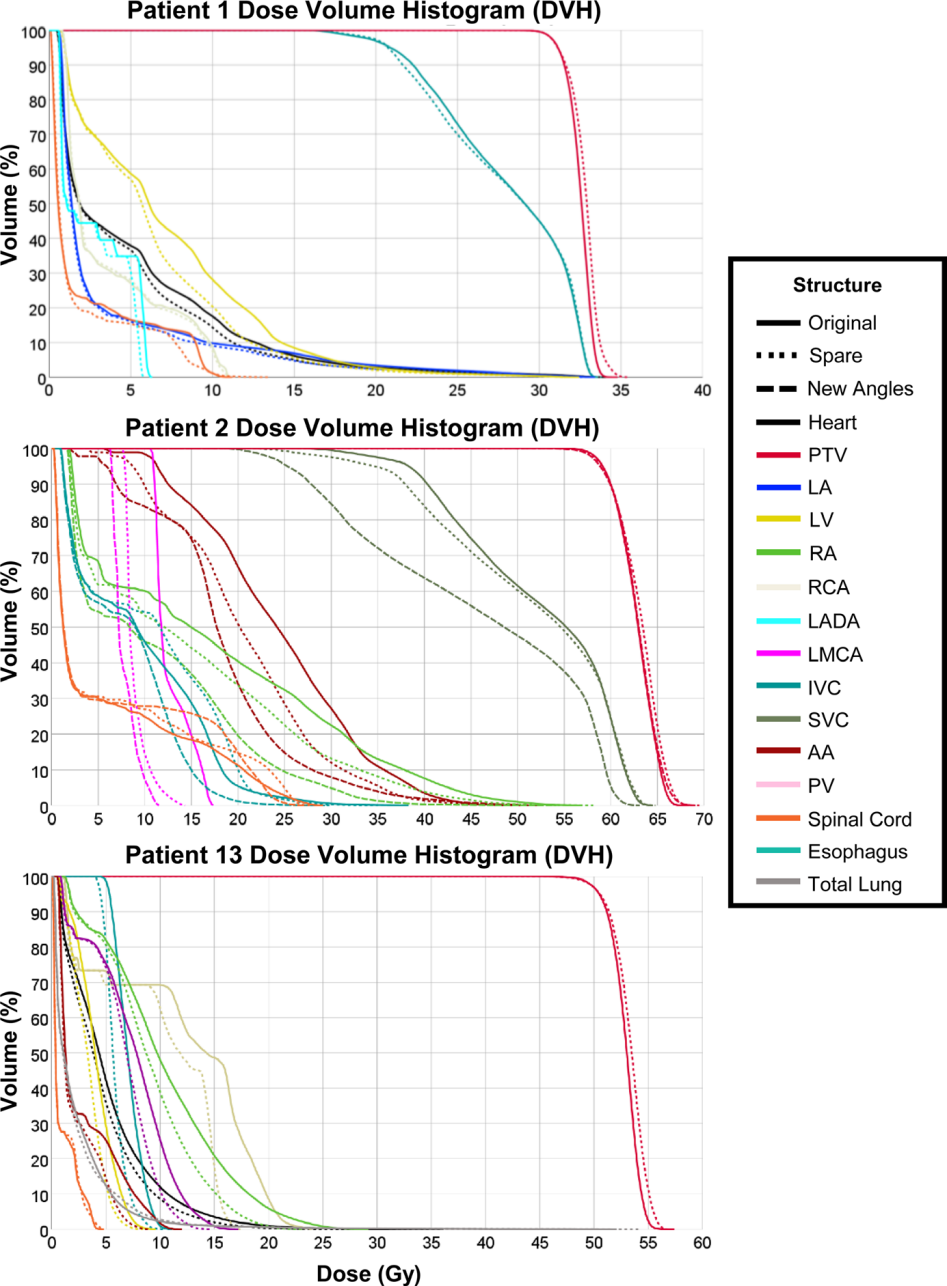
Dose–volume histograms (DVH) for three patients of the least effective cardiac substructure sparing (Patient 1), highly effective sparing (Patient 2), and an average case (Patient 13) showing dose from the original clinical treatment plan and after re‐optimization. The modified beam angle plan is also shown for Patient 2. Abbreviations defined in the text.

Fig. 3 shows dose–volume histograms (DVHs) for three patients selected to represent an example of the least effective cardiac substructure sparing (Patient 1), highly effective sparing (Patient 2), and an average case (Patient 13). Each DVH shows the PTV, involved OARs, and relevant cardiac substructures for both the original clinical treatment plan and the re‐optimized plan. Patient 2 benefited from beam angle modifications, and thus, that plan is represented as well. Fig. 3 highlights that for the patients shown, negligible differences (<1 Gy) were observed for the mean lung dose and D_0.03cc_ to the spinal cord indicating comparable plan quality was achieved even when cardiac substructure sparing was implemented. Radiation doses to the whole heart and total lung (results not shown for all patients) were reduced for all patients after re‐optimization, with even further reductions after beam angles were modified. For Patient 2, the mean esophageal dose decreased by 3.0 Gy from the original clinical plan and 4.5 Gy from the re‐optimized plan after modifying the beam angles, all while reducing the mean dose to the AA, SVC, RA, and LMCA by more than 5 Gy.

**Fig. 4:**
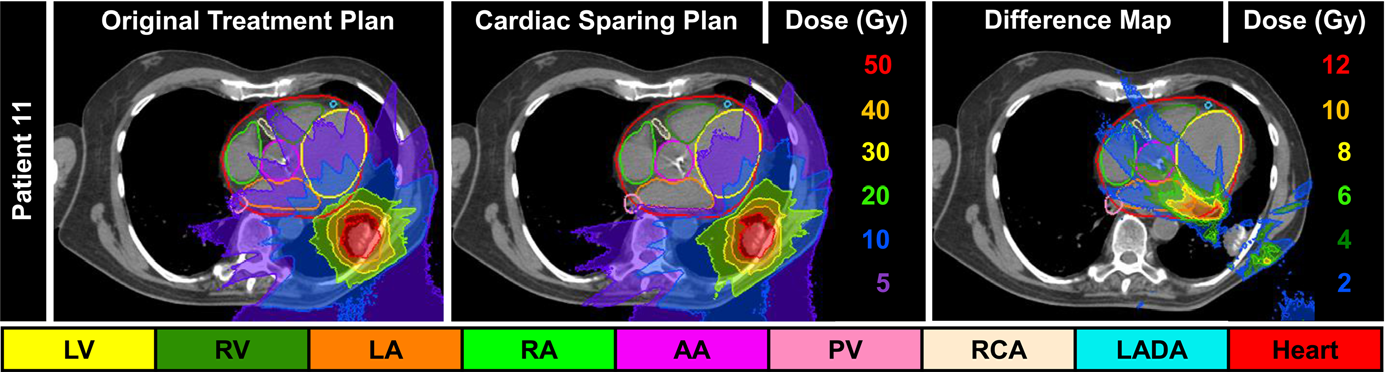
(Left) Initial clinical treatment plan, (middle) corresponding cardiac SPARE treatment plan, and (right) a difference map of initial minus SPARE for Patient 11. Abbreviations defined in the text.

Fig. 4 illustrates the initial clinical treatment plan of a malignant neoplasm of the lower left lung bronchus (left) treated to 48 Gy in four fractions and the corresponding cardiac SPARE treatment plan (right) for Patient 11. This figure highlights cardiac substructure sparing with > 10 Gy reductions in D_0.03cc_ to the LV, LA, and PV. Specifically, note the removal of the 5 and 10 Gy isodose lines from many heart substructures (LA, AA, RA, PV, and RV) after the re‐optimization.

**Fig. 5:**
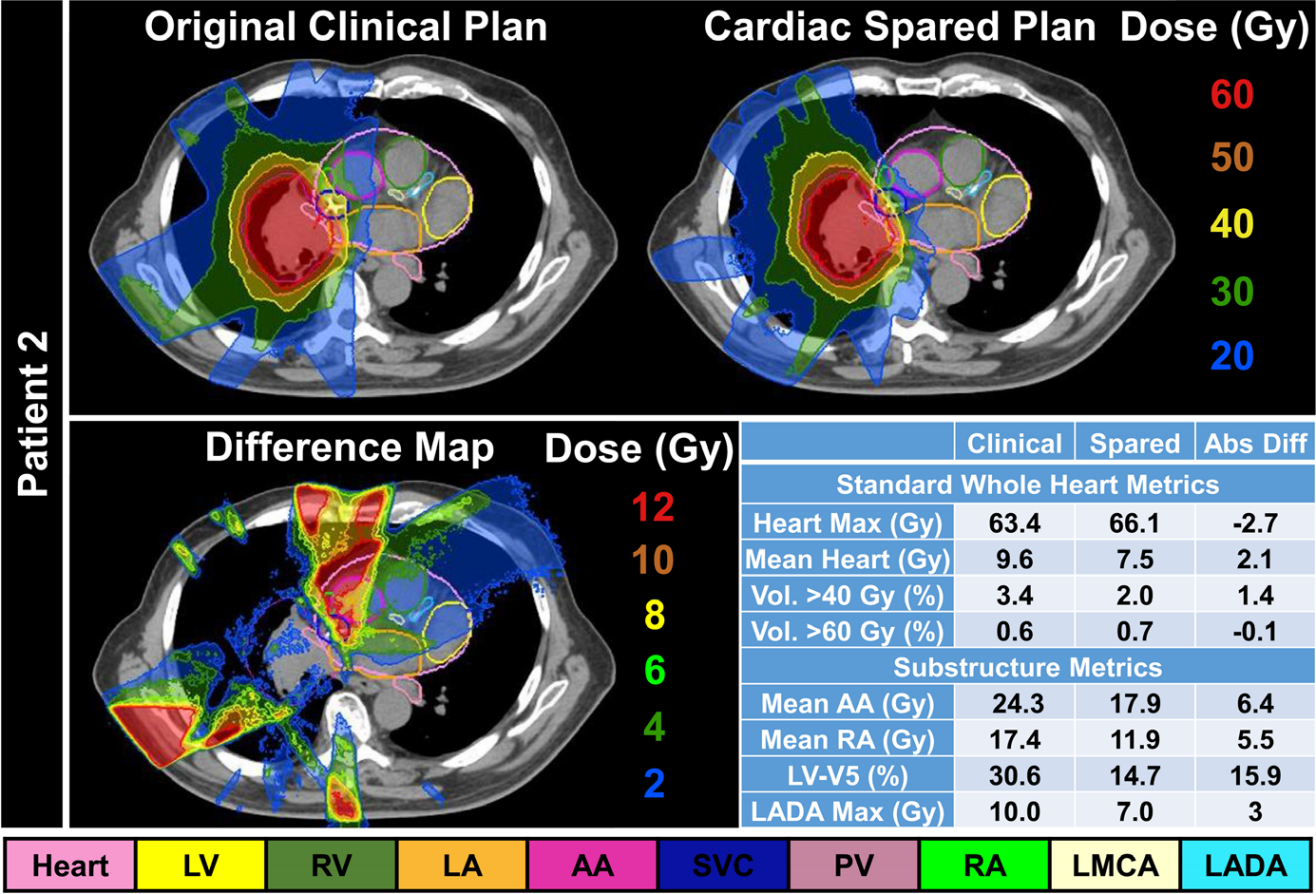
Top row: (Left) Clinically treated plan for an advanced stage lung cancer patient. (Right) Cardiac substructure spared plan. Bottom row: (Left) Dose difference map (clinical less cardiac spared plan) highlighting major dose reductions to cardiac substructures. (Right) Dose metric table showing select standard whole heart dose metrics and substructure metrics. Maximum dose defined as dose to 0.03 cc volume. Abbreviations defined in the text. DVH shown in Fig. 3.

Fig. 5 shows the clinically used radiation treatment plan for Patient 2 (DVH also shown in Fig. 3) that originally met all whole‐heart dose endpoints for a locally advanced lung cancer patient treated to 60 Gy in 20 fractions. Cardiac sparing after re‐optimization is shown with the original clinical treatment plan shown (top left), the cardiac SPARE plan (top right), and the difference map (bottom left). The dose metric table (bottom right) highlights that standard whole heart dose metrics (<3 Gy and <2% absolute difference) do not reflect the local dose deposition that the substructure metrics are able to capture. For example, the LV‐V5 was reduced from 30.6% to 14.7% after re‐optimization. Furthermore, the mean dose to the AA was reduced by ~ 6 Gy and the LADA D_0.03cc_ was reduced below 10 Gy (threshold for coronary artery calcification^9^ presented in Table 1) with sparing.

**Fig. 6:**
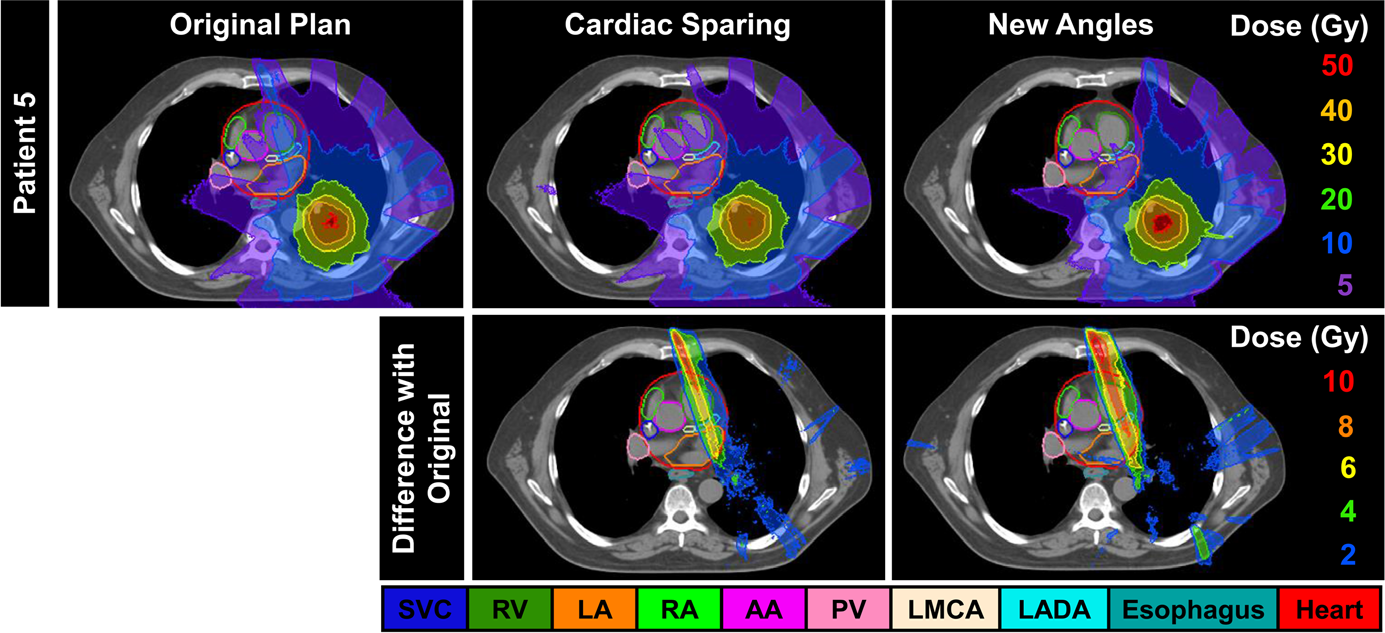
Top row: Original clinical plan (left), re‐optimized SPARE plan (center), and New Angles plan (right) for a patient with a left lung tumor. Bottom row: Difference maps comparing the re‐optimized SPARE plan and the New Angles plan to the original clinical plan. Difference maps are the original plan less the new plan. Abbreviations are defined in the text.

Optimal beam arrangements led to further cardiac substructure dose reduction in 4 patients. Fig. 6 shows the original clinical plan (left), re‐optimized SPARE plan (center), and New Angles plan (right) for Patient 5 who had a left lung cancer treated to 48 Gy in 4 fractions. This figure shows that although there was a slight change for the cardiac substructures after plan re‐optimization (mean reduction in mean dose over all substructures: 0.2 ± 2.1 Gy), increased sparing after beam angle modification was possible (mean reduction in mean dose over all substructures: 1.0 ± 1.4 Gy). For example, the mean dose to the pulmonary vein was only reduced by 0.2 Gy after re‐optimization but was further reduced by another 1.1 Gy after beam angle modification. Moreover, beam angle modification allowed for further sparing of the LADA and LA with mean dose reductions of 0.9 and 0.8 Gy, respectively, as compared to the SPARE plan.

## 4 DISCUSSION

This work introduced cardiac substructures into CT‐based treatment planning incorporating a co‐registered low‐field MRI to quantify potential dosimetric advantages for improved cardiac sparing. This was completed through the retrospective re‐optimization of treatment plans, as well as modifying the original beam angle arrangement to minimize cardiac substructure radiation dose, all while attempting to maintain PTV coverage and continuing to meet clinical endpoints for other critical OARs.

Even though current cooperative trials use volumetric measures based only on MHD endpoints,^29^, ^34^ introducing cardiac substructure segmentation into radiation treatment planning may help better study and define radiation‐induced cardiac injury. Some studies have aimed to investigate the dosimetric impact of different types of therapy on cardiac substructure sparing. A study by Ferris et al.^35^ evaluated cardiac substructure sparing for optimized volumetric modulated arc therapy (VMAT) and intensity modulated proton therapy (IMPT) and found that cardiac‐optimized plans led to statistically significant improvements in mean dose to the chambers, great vessels, and coronary arteries. Our findings agree with Ferris et al. with respect to significant reductions in the maximum dose to the LADA and RV (>4 Gy on average) while maintaining or improving clinical OAR (e.g., lung, esophagus, and spinal cord) constraints and PTV coverage. Likewise, Lester et al.^36^ created re‐optimized VMAT plans to reduce radiation dose to the coronary arteries and cardiac valves.

At present, few studies have integrated cardiac substructures into treatment planning optimization. Ferris et al. evaluated cardiac spared plans using VMAT and IMPT with CT for locally advanced non‐small lung cancer patients with a conventional fractionation to 60 Gy under free‐breathing conditions.^35^ Lester et al. focused on cardiac spared planning for mediastinal lymphomas by incorporating ECG‐gated CT and coronary angiography acquired at deep inspiration breath hold.^36^ These patient populations were different from the present study of 11 out of 16 lung cancer stereotactic body radiation therapy cases (3‐4 fractions) with 7 end‐exhalation, 7 end‐inhalation, 2 free‐breathing to test different conditions. In addition, the present study incorporated a low‐field MRI as an adjunct to treatment planning CTs whereas the Lester et al. study used CT angiography. While MRgRT was employed in this work, the dosimetry strategies of re‐optimization using cardiac substructures and beam angle arrangement modification are applicable to other x‐ray‐based treatment planning modalities as our atlas and deep learning substructure segmentations work on CT‐SIM image inputs. Altogether, the results presented in this work are applicable to a variety of settings, tumor sites, breathing states, and fractionation schedules, which appear promising for future work in cardiac sparing.

We have also shown here that negligible increases in treatment time per fraction and MUs delivered after plan re‐optimization were observed, suggesting similar complexity of the radiation treatment plan. Moreover, even though the modified beam angles plans involved either adding or removing beams in the revised treatment plan, the differences in treatment time per fraction and MUs delivered were negligible (*P* < 0.05). This shows that there will be a negligible practical penalty at the machine for incorporating cardiac substructures in the treatment planning process.

We also found that modifying the beam angle and number of beams used to consider cardiac substructures after the plan had been re‐optimized also had the potential to increase cardiac substructure radiation sparing. However, much like the findings by Lester et al.,^36^ the results were patient specific as lesion location and proximity to the heart and its substructures played a role in if the patient would benefit from plan re‐optimization and beam modification. Patients that benefited from beam angle modification varied in both the number of beams added or removed and in the proximity of the lesion to the heart (i.e., directly adjacent). So, although beam angle modification was shown to provide improvements over solely re‐optimizing the plan for select cases (4/16 cases), re‐optimization alone provided the majority of cardiac substructure sparing, and thus we have shown that simply including substructures in the optimization will provide benefit to a large portion of patients. We found that tumor location also plays a role in the extent a substructure is able to be spared, regardless of plan geometry. For example, the LA for Patient 2 was directly adjacent to the tumor volume yet the mean dose difference after re‐optimization of the LA as shown in Fig. 2 revealed only minor improvement (<1 Gy) was possible. Thus, this suggests that sparing substructures closer to the tumor volume may be difficult although accurately quantifying the dose to substructures still offers value for clinical risk assessment.

It is worth noting that although there was a statistically significant sparing of mean dose to the heart achieved after plan re‐optimization, this may be due to the added weight in the optimizer for when all the substructures are included. However, Fig. 4 highlights that standard whole heart dose metrics were not sensitive to a cardiac sparing treatment planning approach, whereas individual substructure endpoints clearly identified dosimetric, and clinically meaningful gains (i.e., associated with clinical outcomes). Furthermore, the insufficiency of quantifying the MHD alone has been recently affirmed by studies recommending the inclusion of cardiac substructures as RT treatments become more conformal (i.e., intensity modulated RT).^15^, ^37^ For example, the LV‐V5, which has been shown to be more predictive of acute cardiac events than mean heart dose,^6^ was reduced ~15% and the mean dose to the AA was reduced by ~6 Gy, suggesting that with confirmation in a larger cohort, further sparing may offer potential for improved survival.^10^ This underscores the importance of using more sensitive metrics for dose evaluation and not simple whole‐heart evaluations that are currently being implemented.

Respiratory motion was managed via breath‐holding for the majority of the patients. At breath‐hold, there is still the potential impact of cardiac motion which was not accounted for in this study due to not having cardiac‐gated 0.35T MR‐linac images. It has been shown that even under breath‐hold conditions, cardiac substructures may displace ~5 to 7 mm throughout the cardiac cycle.^38^, ^39^ Thus, incorporating a planning organ at risk volume (PRV) representing the variability of the cardiac substructures over a patient’s imaging and treatment course will be the next step of this work. However, as substructure PRV recommendations do not currently exist for each substructure and this study was unable to account for cardiac motion, they were beyond the scope of the current work. In the future, however, this may be possible through the use of van Herk’s formalism,^40^ which was used by Levis et al.^39^ to estimate PRVs for the coronary arteries. While this work was based off of CT‐based treatment planning that enables more widespread applicability to x‐ray‐based approaches, MR‐only treatment planning is gaining popularity, and a future direction includes translating the work to MR‐only plans. Moreover, although the initial segmentation techniques used here could be employed in a variety of treatment modalities that use CT as the treatment planning input, this study may provide a gateway to automatic re‐segmentation and daily adaptive planning with MRgRT in hypofractionated and stereotactic body radiation therapy (SBRT) treatments. An application to adaptive planning would allow the employed cardiac substructure dose sparing measures to be maintained throughout treatment.

Finally, increasing the size of the patient cohort with varied target locations will help identify the patient geometries that will benefit most from cardiac substructure sparing, as discussed above. However, the size of the patient cohort in the current study is consistent with the previously mentioned studies where 7–8 patients were used.^36^, ^39^ An increase of size such as this could be completed through applying this work to a prospective clinical trial, like that of Jacob et al.,^41^ or be applied to multi‐institutional studies, such as the study recently completed by Dess et al.,^42^ and could also help to determine if cardiac substructure dosimetric sparing has an effect on clinical outcomes.

## 5 CONCLUSION

This work applied a multimodality imaging and contouring workflow to showcase the possibility of providing robust dose sparing of cardiac substructures with MRgRT. New treatment plans maintained PTV and OAR doses and did not substantially increase delivery time or required monitor units, suggesting stable plan quality and a negligible increase in plan complexity when cardiac substructure sparing was introduced. This study emphasized how high‐quality cardiac substructure segmentations and sparing plans may be generated at low‐field MRI, which offers strong potential for lower substructure doses at initial planning and the ability to further maintain that condition via daily online MR‐guided adaptive radiation therapy. Validation in a larger cohort with appropriate margins will offer the potential to reduce radiation‐related cardiac toxicities and the dose assessment of currently overlooked radiosensitive substructures.

## AUTHOR CONTRIBUTIONS

Eric D. Morris, PhD: Data analysis, manuscript preparation, and figure preparation Kate Aldridge, CMD: Treatment planning and manuscript review. Ahmed I. Ghanem, MD and Simeng Zhu, MD: Contouring, contour review and manuscript review. Carri K. Glide‐Hurst, PhD, PI of IRB study, study conception, analysis, manuscript, and figure preparation.

## CONFLICT OF INTEREST

The research reported in this publication was partially supported by the National Cancer Institute of the National Institutes of Health under award Number R01 CA204189‐01A1. The content is solely the responsibility of the authors and does not necessarily represent the official views of the National Institutes of Health. Dr. Glide‐Hurst reports research agreements with Modus Medical, ViewRay Inc., and Philips Healthcare unrelated to the current work.
